# A genus-labeled dataset of individual trees outside forests in Baden-Württemberg, Germany

**DOI:** 10.1038/s41597-026-08400-y

**Published:** 2026-09-30

**Authors:** Yulia Grinblat, Heike Tost, Anastasia Benedyk, Andreas Meyer Lindenberg, Somidh Saha, Alexander Zipf, Nir Fulman

**Affiliations:** 1 Heidelberg Institute for Geoinformation Technology: HeiGIT, Heidelberg, Germany; 2 Institute of Geography, GIScience Chair, Heidelberg University, Heidelberg, Germany; 3 Department of Psychiatry and Psychotherapy, Central Institute of Mental Health, Medical Faculty Mannheim/Heidelberg University, Mannheim, Germany; 4 German Center for Mental Health (DZPG), Partner Site Mannheim Heidelberg Ulm, Mannheim, Germany; 5 Research Group Sylvanus, Institute for Technology Assessment and Systems Analysis, Karlsruhe Institute of Technology, Karlsruhe, Germany; 6 Institute of Geography and Geoecology, Karlsruhe Institute of Technology, Karlsruhe, Germany; 7 Heidelberg Center for the Environment (HCE), Heidelberg University, Heidelberg, Germany

## Abstract

Detailed data on individual trees growing outside forests (TOF) are essential for arboriculture, biodiversity assessment, ecosystem services assessment, climate adaptation, and environmental health research. However, existing datasets are often incomplete and seldom provide taxonomic information beyond publicly managed trees in cities of Germany and other countries where maintaining a tree cadaster is a legal requirement. Here, we present for the first time a statewide dataset of individual TOF in the state of Baden-Württemberg, Germany, including tree locations, crown geometry, height, and genus-level labels. We mapped trees from high-resolution multi-spectral aerial imagery and height data using deep learning, with terrestrial LiDAR surveys used to create training labels within the URBORETUM project. The released dataset includes ten genus-level classes, including the aggregate categories “Other Deciduous” and “Coniferous” where assignment from aerial imagery is uncertain. The final ten-class genus model achieved a mean average precision (mAP) of 0.382 at an intersection-over-union (IoU) threshold of 0.5 (mAP@0.5) and a macro-averaged F1 score of 0.627 for taxonomic class assignment, while a separately trained one-class tree detector achieved an mAP@0.5 of 0.647, indicating stronger reliability for crown detection than for taxonomic classification. The final inventory contains 16.3 million predicted trees across the mapped non-forest domain of Baden-Württemberg. Beyond providing a statewide dataset, this work offers a scalable vision and operational blueprint for an evolving tree inventory that can be systematically improved over time through better imagery, richer local reference data, and targeted retraining.

## Background & Summary

Trees growing outside forests (TOF), broadly defined as trees occurring on land not classified as forest or other wooded land^1^, are increasingly recognized as important for sustainability, climate adaptation, and quality of life^2^. They can improve air quality, mitigate heat, support biodiversity, and contribute to aesthetics, recreation, health and wellbeing^3–5^. They can, however, also generate disservices, for example by affecting pollutant dispersion, increasing exposure to allergenic pollen, emitting biogenic volatile organic compounds, or posing safety risks under poor management or extreme weather^6^. Because these benefits and disservices vary strongly among individual trees, recent work has highlighted the need for place-based inventories that capture taxonomic and structural attributes such as diameter, height, and crown size to support planning, management, and species selection so that trade-offs between ecosystem services can be minimized to improve arboriculture^5,7,8^.

Detailed data for TOF remain scarce. Reviews and practitioner surveys show that inventories vary widely in content, completeness, and geographic coverage across communities^9^. Where inventories do exist, access is often limited to municipal authorities rather than the general public^10^. Moreover, most inventories focus on publicly managed street and park trees, whereas trees on private residential parcels, which often account for a substantial share of tree cover, are rarely recorded systematically^10,11^. Field inventories and citizen-science programmes can help fill these gaps, but they are labor-intensive and may suffer from species misidentification or incomplete taxonomic resolution^12^. As a result, many studies and planning efforts still rely on coarse greenness indicators, such as canopy cover, rather than detailed tree-level information^13^.

Very-high-resolution aerial imagery and Light Detection and Ranging (LiDAR) now provide sufficient spatial, spectral, and three-dimensional detail to resolve variation among TOF, even in heterogeneous urban environments^14–17^. Deep-learning methods can use these data to delineate tree crowns and derive tree-level attributes, including structural characteristics and species or broader taxonomic groups, from multi-source remote-sensing data at scale^16,18^. These developments have made it increasingly feasible to generate detailed, city-scale tree inventories from remote sensing rather than relying only on field-based surveys. However, available data still differ substantially among cities in spatial resolution, spectral bands, and acquisition dates, so workflows must often be adapted to local conditions to produce comparable tree-level information products.

**Fig. 1:**
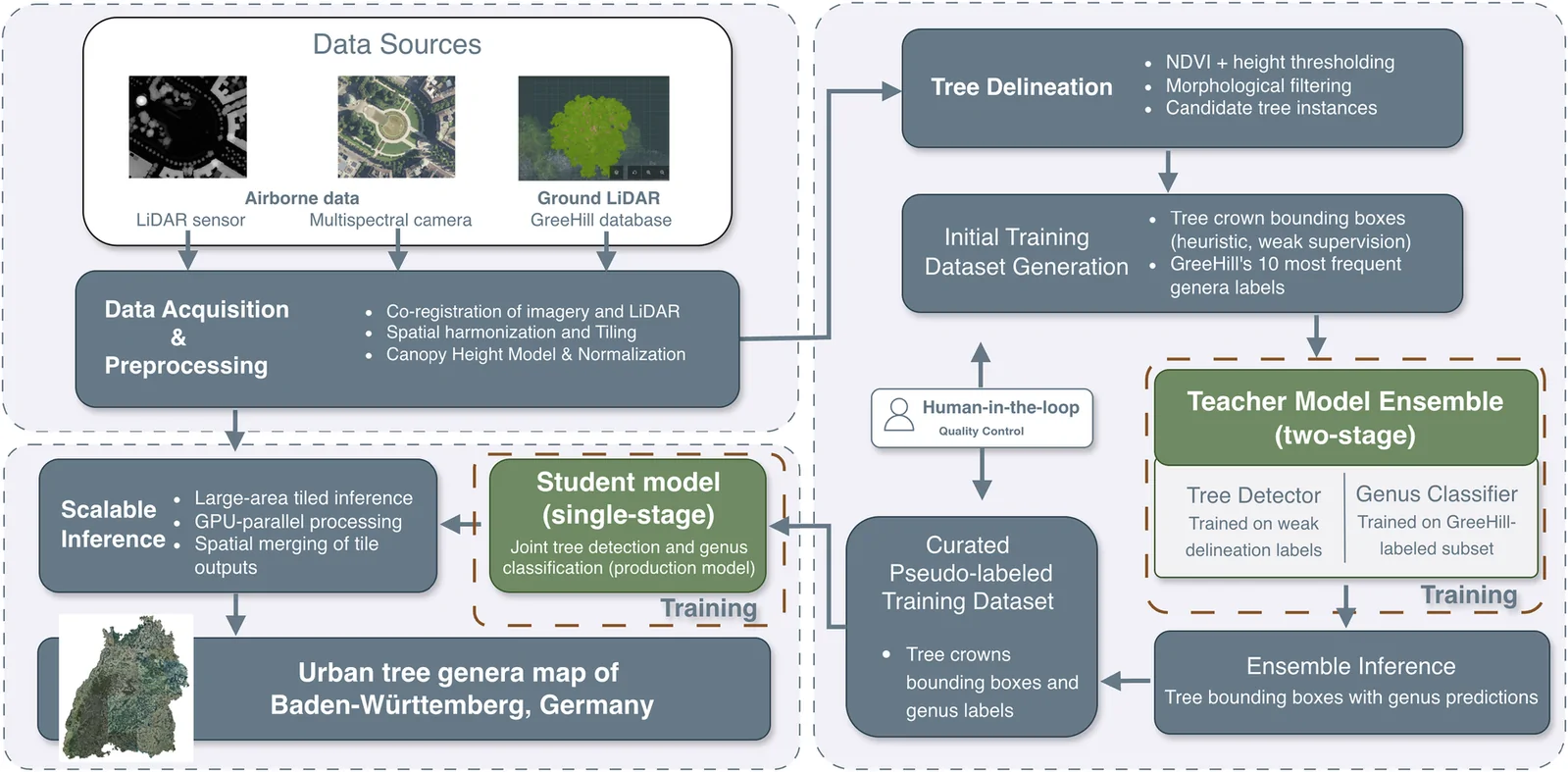
End-to-end workflow schematic (inputs → delineation → teacher ensemble → human curation → student YOLO11l → statewide inference).

Here, we present a statewide genus-level dataset of individual TOF for Baden-Württemberg, Germany, derived from multi-sensor remote sensing and deep learning (Fig. 1). The workflow combines statewide aerial orthophotos and elevation data with terrestrial mobile-mapping reference data from Mannheim, Karlsruhe, and Freiburg, which provide tree locations and genus labels along surveyed road and park corridors. Because these terrestrial reference data are spatially limited, they were not sufficient on their own to train a robust statewide mapping model. We therefore developed a multi-stage workflow in which the initial labeled set was expanded through model-based pseudo-labelling and expert curation, and the resulting annotations were used to train a final multi-class detector. This detector was then applied across the statewide non-forest domain to predict TOF locations and genus. The released data product includes TOF locations, crown geometry, height, and genus-level labels, together with training annotations, metadata, and code.

## Methods

### Study area and scope

**Fig. 2:**
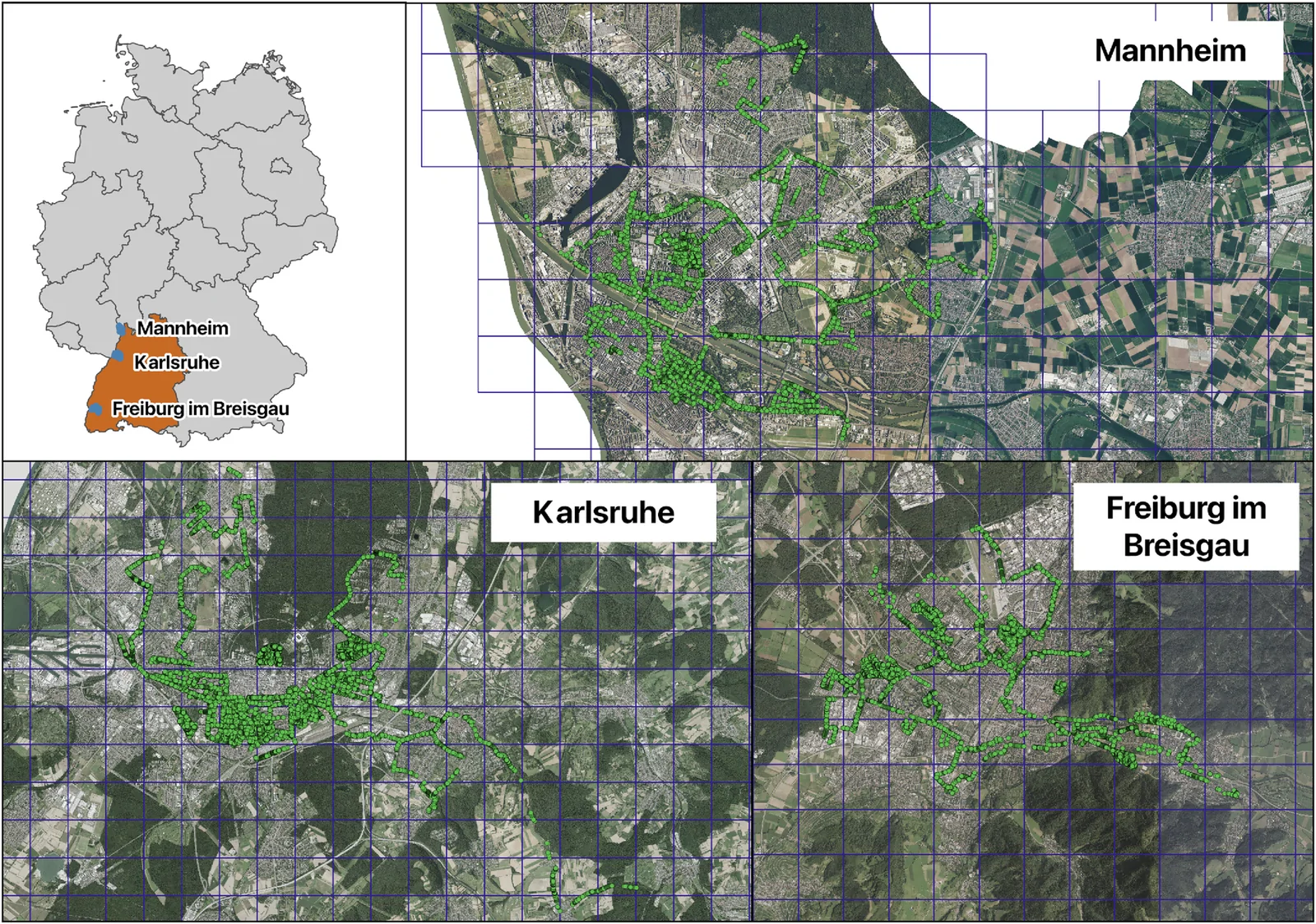
Study area: Baden-Württemberg urban mask, tile grid, and locations of Greehill’s LiDAR reference survey in three cities: Mannheim, Karlsruhe, Freiburg.

Our dataset covers Baden-Württemberg, a state in southwest Germany with an area of 35,700 km^2^ and a population of 11.3 million^19^ (Fig. 2). The region includes large metropolitan areas such as Stuttgart, Mannheim, and Karlsruhe, as well as many smaller towns embedded in a landscape characterized by extensive forest cover and intensively used agricultural land. Consistent with the general definition of TOF given above, the dataset is restricted to the non-forest areas of Baden-Württemberg. The mapping scope includes residential and commercial areas, transportation corridors, agricultural areas with scattered trees, and urban green spaces on both publicly managed and privately owned land. Within this scope, an individual tree refers to a spatially distinct tree canopy.

The dataset focuses on the dominant tree groups found in Baden-Württemberg’s settled and other non-forest areas, at a taxonomic resolution that can be robustly distinguished from aerial imagery. Plant nomenclature follows the International Code of Nomenclature for algae, fungi, and plants (Madrid Code)^20^. Because the taxonomic labels used in this study are assigned at genus rank, genus names are italicized and reported without species epithets or nomenclatural authorities. Urban tree inventories and planning surveys indicate that broadleaf genera such as *Acer*, *Carpinus*, *Quercus*, *Tilia*, and *Prunus* are among the most common groups in major cities^21–23^. Non-forest landscapes in the state also include conifer plantings and traditional orchard meadows with scattered fruit trees of multiple species^24^.

### Data sources

#### Terrestrial LiDAR surveys

We used ground-based mobile mapping surveys acquired by Greehill Deutschland GmbH^25^ in July 2024 in Mannheim, Karlsruhe, and Freiburg, Germany (Fig. 2). The surveys were conducted in areas including inner-city streets, residential neighborhoods, and park and road corridors. Greehill collected three-dimensional point clouds and street-level imagery using vehicle-mounted LiDAR and imaging sensors. These data were processed by the company to generate tree-level inventory records, from which we obtained records for 42,194 trees: 10,087 in Mannheim, 20,258 in Karlsruhe, and 11,849 in Freiburg. In this study, we used only the geographic position and genus label of each tree to derive training data for genus-level mapping from aerial remote sensing. Only the derived training labels used in this study are shared with the dataset described here.

The statewide geospatial input data used in this study were obtained through the Open GeoData program of the Landesamt für Geoinformation und Landentwicklung Baden-Württemberg (LGL BW)^26^. All datasets were provided in the ETRS89 / UTM coordinate reference system.

#### Aerial orthophotos

We used the 2024 digital orthophotos DOP20 for Baden-Württemberg^27^. DOP20 comprises geometrically corrected Red-Green-Blue (RGB), and near-infrared (NIR), orthophotos represented as raster layers, with a ground resolution of 20 cm, and is distributed in 1 km × 1 km tiles.

#### Digital terrain model

We used the LiDAR-derived digital terrain model DTM1 (distributed by LGL as DGM1)^28^, a raster terrain model with 1 m grid spacing representing the ground surface without vegetation, buildings, or other above-ground structures. DTM1 was generated from airborne laser-scanning data acquired between 2016 and 2021, with a reported vertical accuracy of approximately 15 cm.

#### Digital surface model

We used the digital surface model distributed by LGL under the original German product name DOM1 (hereafter DSM1)^29^. This raster model has a 1 m grid spacing and represents the elevation of the land surface, including vegetation and built structures. DSM1 was generated from statewide LiDAR data acquired between 2016 and 2021, so acquisition dates may vary among tiles. Reported vertical accuracy is approximately 15 cm for solid, vegetation-free surfaces; the data provider notes that larger deviations may occur over vegetation and tall, narrow objects, which is relevant given this dataset’s focus on tree crowns. We combined DSM1 with DTM1 to derive a normalized digital surface model A (nDSM A).

#### Normalized digital surface model B (nDSM B)

We used the LGL normalized digital surface model (distributed by LGL as nDOM; hereafter nDSM B)^30^. This product has a spatial resolution of 1 m and provides complete statewide coverage. It combines a DSM derived from 2024 imagery with the DTM1 terrain model acquired between 2016 and 2021. Its main advantage is that the surface information is temporally aligned with the 2024 DOP20 aerial imagery. However, because the DSM is image-based rather than LiDAR-based, its vertical accuracy is lower than that of nDSM A, at approximately 40–60 cm.

#### Ancillary geodata

We used the Basic Digital Landscape Model (Digitales Basislandschaftsmodell, Basis-DLM)^31^, a statewide two-dimensional topographic vector dataset, to delineate the state boundary and operationally define the non-forest mapping domain. Within the Basis-DLM, land-cover objects are represented by polygons classified according to the ATKIS object catalogue^32^. Forest areas were identified in the vegetation layer *AX_Wald* (*veg02_f.shp)*. To distinguish settlement from non-settlement TOF, we used the official ATKIS settlement layer *AX_Ortslage* (*sie01_f.shp*). The *AX_Ortslage* layer represents contiguous settlement areas and includes residential, commercial, industrial, transport, recreational, water, and associated vegetation areas. The Baden-Württemberg state boundary and municipality boundaries used for spatial aggregation were obtained from the administrative-area layer *geb01_f.shp*, using the ATKIS object types *AX_Gebiet_Bundesland* and *AX_KommunalesGebiet*, respectively.

### Preprocessing

#### Co-registration of imagery and elevation data

We aligned the raster layers to a common grid for joint analysis and, where necessary, corrected small positional offsets between the orthophotos and elevation data by applying sub-pixel translations. DSM1 and DTM1 were resampled from 1 m to the 0.2 m ground resolution of the orthophotos using bilinear interpolation so that each image pixel had a corresponding height value. All raster layers were then divided into a regular 1 km × 1 km tile grid matching the orthophoto layout and cropped to the Baden-Württemberg boundary and a non-forest mask derived from the Basis-DLM.

#### Normalized surface model A

We derived above-ground height by subtracting the digital terrain model DTM1 from the digital surface model DSM1, resulting in nDSM A. This raster represents the height of vegetation, buildings, and other above-ground objects relative to the ground surface. nDSM A was smoothed with a small kernel to reduce local noise while retaining crown structure, and values below a minimum height threshold were set to zero to suppress ground artefacts.

#### Multi-channel input stacks

**Fig. 3:**
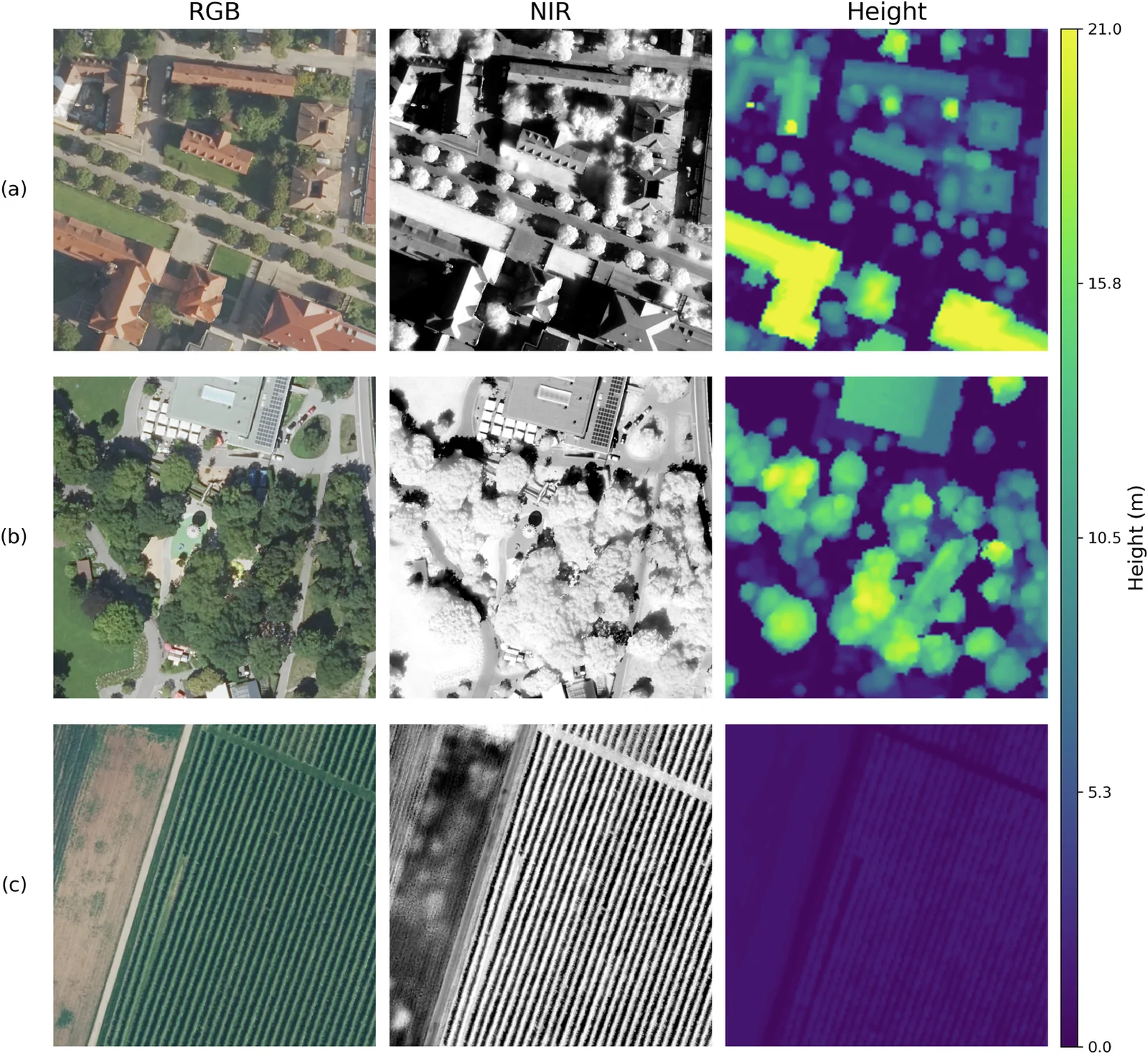
Subtiles with 5 channels 640 × 640 px: (**a**) urban, (**b**) urban greenspace and (**c**) negative cases.

We combined the rasters into five-channel image stacks per tile, consisting of RGB, NIR, and above-ground height (nDSM B). As part of this process, the height rasters were aligned to the 0.2 m orthophoto grid and encoded as unsigned 8-bit channels using fixed global scaling bounds of 0 and 80 m. Thus, a stored value of 0 corresponds to 0 m and a value of 255 corresponds to 80 m. The use of fixed rather than tile-specific scaling preserved comparability of the height channel across training, validation, test, and statewide inference tiles. These five-channel stacks were then used as the standard input format for teacher-stage pseudo-label generation and for training and statewide application of the final detection and classification model (Fig. 3).

#### Genus label scheme

**Fig. 4:**
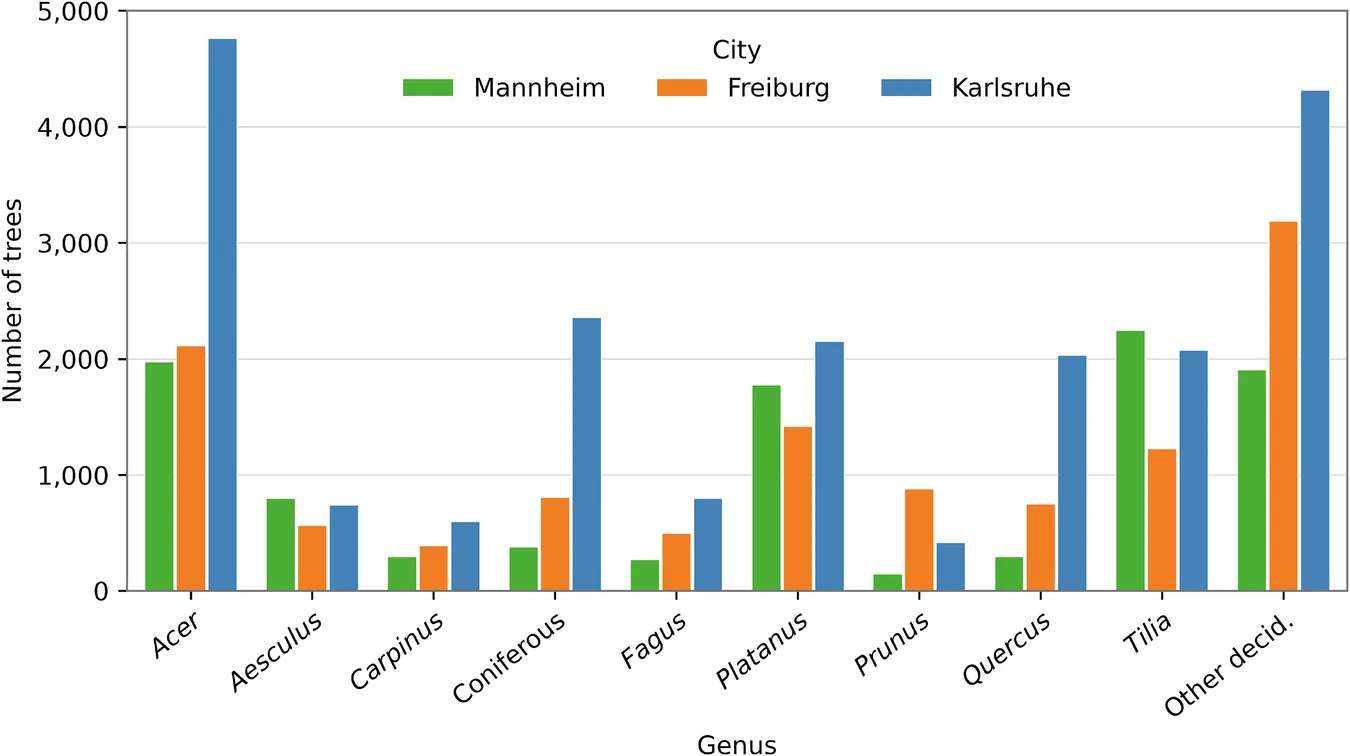
Genus class distribution in the curated training set (including “Other Deciduous” and “Coniferous”).

We defined the label set for this study to represent the dominant tree groups in Baden-Württemberg’s settled and other non-forest areas while remaining compatible with the taxonomic resolution achievable from aerial imagery. The classification task comprised ten classes: *Acer*, *Aesculus*, *Carpinus*, *Fagus*, *Platanus*, *Prunus*, *Quercus*, *Tilia*, “Coniferous”, and “Other Deciduous”. Coniferous trees were included as a separate class because they can generally be distinguished clearly from broadleaf trees in aerial imagery. The two aggregate classes (“Coniferous” and “Other Deciduous”) were used where genus-level assignment from aerial imagery was uncertain (see Study area and scope; Fig. 4).

### Generation of training data via a teacher ensemble

The available reference inventory contains 42,194 trees with geographic positions, crown-size information, and genus labels. However, because the surveys were concentrated along roads and urban corridors, the inventory does not represent the full range of spatial contexts across the wider mapping region. Training an object-detection model for statewide application therefore required a larger and more spatially extensive set of tree annotations containing both crown-extent and genus information.

We therefore generated a larger training dataset. First, we delineated tree crowns in 1,568 Mannheim subtiles intersecting the reference surveys using vegetation and height information. The resulting crown boxes were manually corrected and used as weak labels to train a one-class Faster R-CNN detector. For genus classification, a ResNet was trained in parallel on crown-centred image patches from the inventory of 42,194 reference trees using their recorded genus labels. The two trained models were then applied jointly to the 143 selected 1 km × 1 km parent tiles intersecting the Greehill surveys in Mannheim, Karlsruhe, and Freiburg to generate crown-box and genus predictions. These were subsequently corrected by human annotators to produce the curated training dataset for the student model.

#### Initial weak-label generation by tree delineation

To generate initial weak labels for tree localization, we delineated candidate tree crowns within 1,568 Mannheim subtiles that intersected the Greehill survey paths. We calculated the normalized difference vegetation index (NDVI) from the red and NIR bands of the DOP20 orthophotos and retained pixels with NDVI ≥ 0.3 to produce vegetation masks. Within these masks, we then retained only pixels with an above-ground height ≥2 m in nDSM A, thereby excluding low vegetation and ground-level artefacts. Consequently, trees whose visible crowns did not extend above this threshold were not represented in the initial crown delineation. Smaller trees exceeding the threshold could be delineated only when their crowns were visible from above and spatially separable from neighboring crowns; fully occluded understory crowns could not be resolved from the aerial data.

To separate individual crowns within the resulting tall-vegetation masks, we identified local maxima in nDSM A as candidate crown peaks and applied watershed segmentation to partition connected canopy areas into individual crown polygons. These polygons were then converted into bounding boxes. The resulting boxes were manually reviewed to remove non-tree artefacts, including vegetation on rooftops, and to correct segmentation errors associated with complex or overlapping crowns. The reviewed bounding boxes constituted the weak localization labels used to train the teacher detector.

nDSM A was used for this delineation because its LiDAR-derived height surface provided more reliable local height differences, supporting the identification of crown peaks and the separation of neighboring tree crowns.

#### Teacher-stage pseudo-label generation

Both teacher models used the five-channel RGB, NIR, height (nDSM B) imagery described above. For tree localization, a one-class Faster R-CNN detector^33^ was trained on the 1,568 640 × 640-pixel, five-channel Mannheim subtiles using the reviewed weak crown boxes generated in the preceding delineation step. For genus classification, ResNet-50 and ResNet-101 classifiers were trained on crown-centered five-channel image patches derived from the reference inventory crown size and resized to 128 × 128 pixels. ResNet-101 achieved the best validation performance and was therefore used in the teacher ensemble.

The Faster R-CNN detector and ResNet-101 classifier were then applied jointly across the 143 selected 1 km × 1 km parent tiles intersecting the Greehill surveys in Mannheim, Karlsruhe, and Freiburg. Faster R-CNN first generated candidate tree-crown bounding boxes. For each detected box, the corresponding five-channel image patch was extracted, resized to 128 × 128 pixels, and classified by ResNet-101. A detection and its assigned genus class were retained together as a box-and-class pseudo-label only when the detector confidence and classifier confidence both exceeded their predefined thresholds.

#### Human-in-the-loop (HITL) curation

**Fig. 5:**
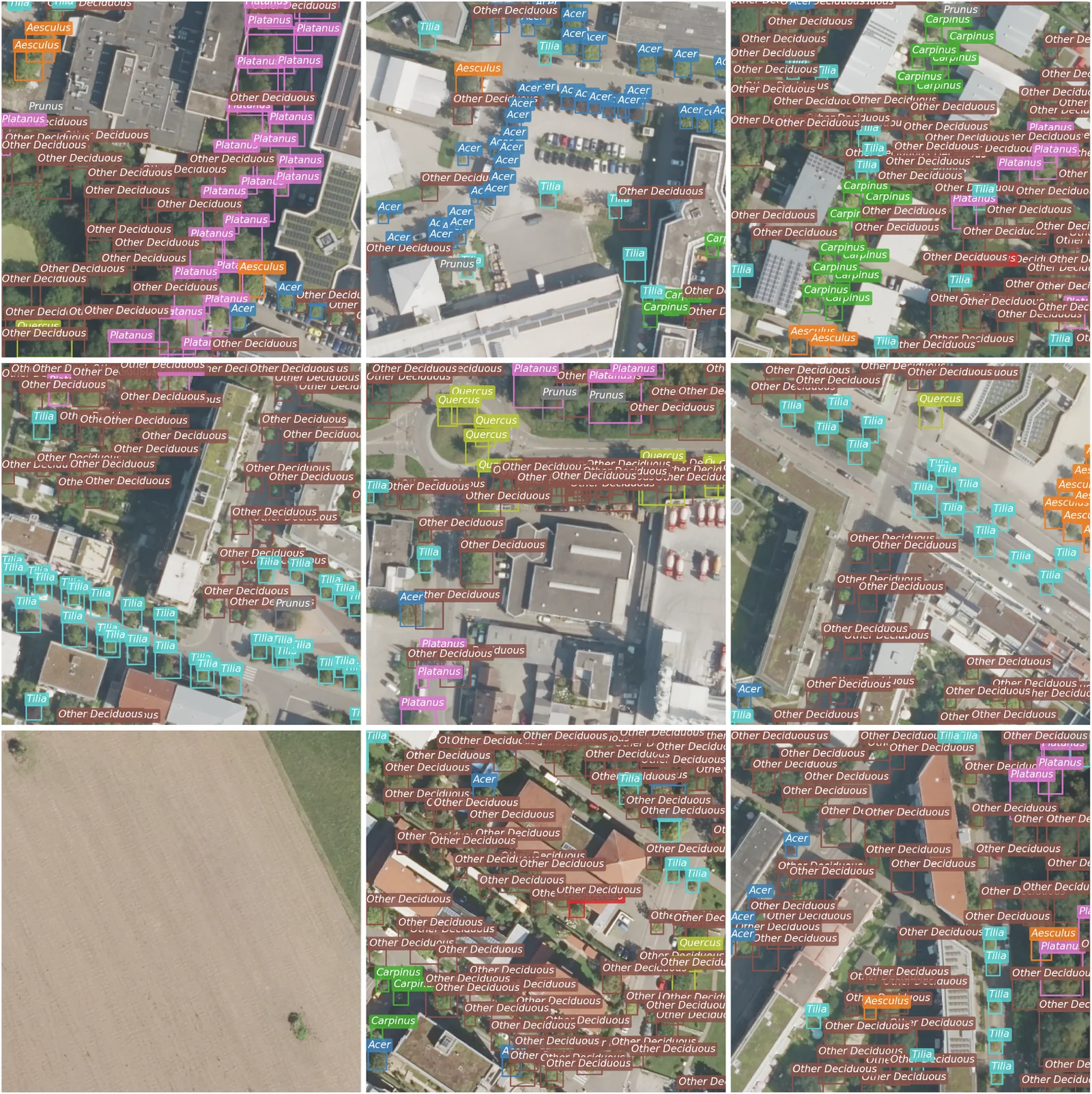
Training dataset with the curated pseudo labels on subtiles 640×640 px.

The teacher-generated pseudo-labels were organized into 2,965 overlapping image subtiles selected for manual curation. Because adjacent subtiles overlapped, some trees were represented by annotations in more than one subtile; these duplicate annotations were identified and removed before manual review. The remaining teacher predictions were then matched against the Greehill terrestrial survey inventory. For matched trees, the teacher-derived bounding box and genus prediction were replaced by the survey-derived crown geometry and genus label, whereas surveyed trees without a corresponding teacher detection were added directly. The resulting annotation set was loaded into QGIS Desktop^34^ and divided into two spatially non-overlapping review subsets. Each subset was assigned to one of two annotators with expertise in remote-sensing image interpretation. The review involved correcting erroneous genus labels, adjusting clearly mis-sized crown boxes, separating boxes that incorrectly covered multiple crowns, and removing obvious false positives. The corrected annotations were compiled into the curated training dataset used in the next stage (Fig. 5). Human curation was confined to the training dataset and was not applied to the final statewide predictions. Because the two annotators reviewed different spatial subsets, no annotation was independently assessed by both annotators. Consequently, no formal disagreement-resolution procedure was applied, and inter-annotator agreement could not be calculated. Further details of the curation protocol are provided in Supplementary Note 1.

### Statewide inventory model

We trained a single-stage student detector to predict both tree locations and genus labels from five-channel raster stacks, using the curated pseudo-labeled dataset produced by the teacher ensemble for supervision. The model was implemented in the Ultralytics YOLO framework^35^ using the YOLO11l architecture. We selected this high-capacity architecture for dense small-object detection and ten-class genus classification because statewide inference was performed offline and therefore inference speed was not a primary constraint. The YOLO11l architecture variant was fixed before the ablation experiments; only the input channels, input resolution, and genus-label scheme were varied. A systematic comparison of different YOLO11 size variants was outside the scope of this study. We compared RGB, RGB + NIR, and RGB + NIR + height inputs, multiple input resolutions, and five- and ten-class genus schemes, and selected the final configuration for statewide inference based on predictive performance and the level of taxonomic detail retained. Detailed results of these configuration comparisons are provided in Supplementary Note 2.

The network was initialized from weights pretrained on the Microsoft COCO dataset^36^, and subsequently adapted using the VisDrone aerial-imagery dataset^37^. This intermediate training stage exposed the model to aerial viewing geometry and object scales more similar to those in the present application before fine-tuning on the curated tree dataset. For the final five-channel configuration, the additional input channels required separate initialization: the NIR-channel weights were initialized from the pretrained red-channel weights, whereas the height-channel weights were initialized using Kaiming initialization. This retained the pretrained RGB representations while providing suitable initial weights for the additional spectral and structural inputs.

### Evaluation design and data partitioning

The curated annotation dataset used to train and evaluate the statewide inventory model was derived from 1 km × 1 km parent tiles intersecting the Greehill survey areas in Mannheim, Karlsruhe, and Freiburg. Subtiles without annotated trees were retained as negative samples to represent challenging non-tree environments, including agricultural land, water bodies, railway corridors, and airports. To assess performance within the represented survey domain and under geographic transfer, two complementary partitioning strategies were used (Table 1).

**Table 1:** Distribution of curated annotations and subtiles by city and partitioning strategy.

City	Strategy 1: Tile-based Split (% of img)			Strategy 2: City-to-City Split (% of img)			Total subtiles (*Nimg*)	Annotation Instances (*n*)
	Train	Val	Test	Train	Val	Test		
Mannheim	77.7	15.7	6.6	100	0	0	961	48,129
Karlsruhe	84.0	8.9	7.1	100	0	0	1,205	68,334
Freiburg	90.5	4.9	4.6	0	62.1	37.9	799	41,623
Total	83.7	10.0	6.3	73.1	16.7	10.2	2,965	158,086

Abbreviations: img, images; Nimg, total number of subtiles/images; n, total number of annotated tree instances.

#### Tile-based spatial split

Parent-tiles from Mannheim, Karlsruhe, and Freiburg were pooled and assigned to training, validation, and test partitions before subtile extraction. Each parent-tile was assigned to only one partition. The tiles were subsequently divided into 640 × 640 pixel subtiles with 20% overlap within each partition to reduce edge artefacts during sliding-window processing, while preventing spatial overlap across partitions. The resulting subtile dataset comprised 2,482 training subtiles containing 136,208 annotated tree instances, 297 validation subtiles containing 12,836 instances, and 186 test subtiles containing 9,042 instances, corresponding to 83.7% training, 10% validation, and 6.3% testing of the subtiles, respectively. The test partition was used for evaluation of the selected model configuration.

#### City-to-city split

To assess whether the model transferred to an urban area that contributed no training data, Mannheim and Karlsruhe were used for training, while Freiburg was reserved for validation and testing. Unlike the pooled tile-based split, this design provides a geographically independent holdout. This is relevant to statewide application because most mapped areas lie outside the reference surveys. Freiburg represents a new combination of tree-genus composition, urban form, planting patterns, surrounding surfaces, and image appearance^38^. The experiment measures the combined effect of these differences on performance and as such, cannot attribute it to any single factor. Partitioning was performed at the parent-tile level before subtile extraction, with overlap allowed only within each partition. All subtiles from Mannheim and Karlsruhe were assigned to the training set, whereas Freiburg subtiles were divided between validation and testing.

### Evaluation metrics

Because individual tree crowns are irregular, partially overlapping, and often ambiguously delineated in aerial imagery, evaluation was not restricted to the conventional intersection over union (IoU) threshold of 0.5. Additional thresholds of 0.3 and 0.4 were considered to provide a more ecologically meaningful assessment of crown localization and inventory completeness^39^.

The Faster R-CNN teacher detector was evaluated using precision, recall, F1-score, and average IoU across multiple IoU thresholds. Because the detector is one-class, performance is summarized using these metrics rather than class-averaged mean average precision (mAP).

The ResNet-based teacher classifier was evaluated using precision, recall, F1-score, and macro F1, where macro F1 accounts for class imbalance among the target genera.

For the student model, experiments were conducted in both a one-class tree-detection setting and a ten-class genus-detection setting. Detection performance was evaluated using mAP at IoU thresholds of 0.3, 0.4, and 0.5, together with the COCO-style summary metric mAP@0.5–0.95^36^. Here, the “@” symbol denotes the IoU threshold used for evaluation; thus, mAP@0.5 represents mAP at an IoU threshold of 0.5, whereas mAP@0.5–0.95 is averaged across IoU thresholds from 0.5 to 0.95 in increments of 0.05. Precision, recall, and F1-score are additionally reported for tree-detection experiments, while macro F1 is used for genus classification.

### Generation of statewide predictions

After training, the final YOLO11l student detector was applied to statewide five-channel raster stacks covering the TOF mapping domain of Baden-Württemberg. Inference was conducted tile-by-tile over 35,674 1 km × 1 km tiles. A global detection-confidence threshold of 0.30 was applied to all predictions.

Statewide inference produced an apparently implausible proportion of predictions assigned to Acer. We therefore introduced an empirical post-processing rule: Acer predictions with confidence below 0.50 were retained as tree detections but reassigned to the broader “Other Deciduous” category, whereas predictions with confidence greater than or equal to 0.50 retained the Acer label. This threshold was not selected through formal optimization. The class-weighting experiment is reported in Supplementary Note 2.5.

The final outputs were exported as vector features containing predicted box geometry, genus label, and model confidence for each mapped tree.

### Calculation of municipality-level tree indicators

Final tree predictions were summarized at the municipality level to derive indicators of tree composition. Tree density was calculated as the number of predicted trees per km^2^ of valid mapped area,

$$D=\frac{{N}_{{trees}}}{{A}_{{mapped}}}$$

where ${N}_{{trees}}$ is the number of predicted trees and ${A}_{{mapped}}$ is the non-forest area of the municipality. To characterize compositional heterogeneity, genus richness was defined as the number of unique predicted genera within each municipality, and normalized Shannon diversity was calculated for 100 × 100 m grid cells as

$${H}_{{norm}}=\frac{-\mathop{\sum }\limits_{i=1}^{n}{p}_{i}{\log }({p}_{i})}{{\log }(n)}$$

where *pi* denotes the proportion of trees assigned to genus *i* within the cell, and *n* is the total number of genera considered in the classification scheme. The resulting normalized diversity values were then aggregated to municipality level by calculating the mean across all grid cells within each municipality.

### Computational environment and training configuration

Model training and inference were conducted on the Helix high-performance computing cluster at Heidelberg University. The workflow used NVIDIA A40 and A100 GPUs with 40–60 GB VRAM under CUDA 12.4. All models were implemented in Python 3.11.14 using PyTorch 2.5.1, torchvision 0.20.1, and Ultralytics 8.3.221. The teacher-stage Faster R-CNN detector and ResNet classifiers were built with standard PyTorch and torchvision components, whereas the student detector used the Ultralytics YOLO11 framework, adapted to accept five input channels rather than the default three-channel RGB input. Raster preprocessing and feature generation, including reprojection, resampling, tiling, nDSM computation, NDVI calculation, and crown delineation, were implemented in the same environment using GDAL, rasterio, NumPy, SciPy, and scikit-image.

## Data Records

The dataset has been deposited in the heiDATA repository at https://doi.org/10.11588/DATA/MKZPUY^40^ and comprises the six records described below.

### Record 1: Baden-Württemberg non-forest tree genus inventory (primary product)

The main data product is an inventory of TOF for all non-forest areas of Baden-Württemberg. It comprises polygons representing individual tree crowns and their centroids for over 16 million trees across ten genus-level classes, stored as features with geometry and attributes in GeoPackage format. For each tree, we provide a unique ID, predicted genus label (10-class scheme), model confidence, crown area, crown height, the ID of the 1 km × 1 km tile in which it was detected in the statewide tiling grid, key acquisition metadata (orthophoto year, DSM1 year), and a flag indicating whether the prediction is located within or outside a contiguous settlement area. This flag was derived from the ATKIS settlement layer *AX_Ortslage (sie01_f.shp)*.

### Record 2: Reference inventory derived from Greehill ground surveys

The reference dataset derived from mobile mapping surveys conducted by Greehill in July 2024 is provided for three cities: Mannheim, Karlsruhe, and Freiburg. It is provided as part of the deposited dataset^40^. The original surveys comprise tree-level inventory information generated from vehicle-based LiDAR. For this data release, we include only the subset of attributes used in this study: tree coordinates, genus labels, and selected crown-related attributes required for training data generation and evaluation. The released dataset contains 42,194 reference trees and is provided in CSV format. All coordinates are supplied in the same coordinate reference system as the other spatial datasets.

### Record 3: Training and validation annotations (curated benchmark subset)

We provide the curated annotations and image subtiles used to train and evaluate the student model. After duplicate removal and HITL curation, the vector annotation layer contained 117,678 curated tree annotations. Assignment of these annotations to overlapping subtiles yielded 158,086 annotation instances across 2,965 five-channel images.

The deposited files comprise curated_pseudo_labels.gpkg, containing the vector annotations and genus labels; subtiles_rgbih_640_20.zip, containing the five-channel RGB, NIR, and height GeoTIFF subtiles together with the corresponding annotations in YOLO-format.txt files. The image–label pairs are organised according to the training, validation, and test partitions used for model development and evaluation.

Partition assignments are provided at both parent-tile and subtile levels in tiles_split.txt and subtiles_split.txt, including the training, validation, and test splits used for model development and evaluation.

### Record 4: Supporting spatial data, metadata, documentation, and code

The supporting material includes the official LGL 1 km × 1 km tile grid used by the workflow (lgl_bw_tiles.gpkg), the repository README, the external-source and licence table (_sources.csv), and a data-record description document containing file- and field-level metadata.

The analysis code is archived in the deposit as tree-genera-mapping-main.zip and is also maintained at https://github.com/GIScience/tree-genera-mapping. The archived copy provides a fixed version corresponding to the workflow described here, including scripts and configuration material for preprocessing, model training, evaluation, post-processing, statewide inference, and derived-indicator generation.

### Record 5: Trained YOLO11l model weights

The repository includes two separately trained YOLO11l model checkpoints in the weights/ directory. The file yolo11l_tree_genus.pt contains the final ten-class genus-labelled detector used to generate the statewide inventory. The file yolo11l_tree.pt contains an auxiliary one-class tree-crown detector provided to benchmark crown detection independently of genus attribution.

The repository documentation records the input-channel order, class identifiers, confidence thresholds, height-channel encoding, and software requirements. The one-class checkpoint was not used to assign genus labels in the released statewide inventory.

### Record 6: Aggregated tree indicators at municipality level

This record contains bw_tree_indicators.gpkg, which provides municipality-level tree count, valid mapped area, tree density, class richness, and mean normalized Shannon diversity as attributes of municipality-centroid points. Mean normalized Shannon diversity was calculated from intermediate 100 × 100 m grid-cell values as described in the Methods; these intermediate grid-cell data are not included in the deposit.

These indicators are derived from the released individual-tree inventory and were not independently validated against municipality-level reference data. Their reliability therefore depends on the accuracy of the underlying tree detections, class assignments, mapped-area estimates, and aggregation procedures.

## Technical Validation

This section assesses the quality of two principal data products released with this Data Descriptor: the curated annotation layer described in Record 3 and the statewide tree inventory described in Record 1. The curation protocol is documented in Supplementary Note 1, whereas the model-development comparisons used to select the architecture, input channels, input resolution, taxonomic configuration, and training procedure are reported in Supplementary Note 2.

### Validation scope

Quantitative validation was restricted to held-out image subtiles from selected Greehill survey areas in Mannheim, Karlsruhe, and Freiburg. These surveys covered streets, residential neighbourhoods, parks, and road corridors but did not provide complete coverage of the three cities or a representative statewide validation sample. The results reported below therefore characterize reliability within the environmental and imaging conditions represented by these survey areas and should not be interpreted as statewide accuracy estimates.

### Validation of the Teacher-generated annotations

#### Crown localization

Performance of the Faster R-CNN was substantially higher at IoU@0.3 (F1 = 0.707) than at IoU@0.5 (F1 = 0.576) (Table 2). This decline likely reflects the irregular and often overlapping boundaries of tree crowns, which make exact agreement between predicted and human-delineated boxes difficult. Given these characteristics, the lower IoU threshold better captures detection completeness for ecological inventory applications^40^. Consistent with this interpretation, recall was higher at IoU 0.3 (0.788), indicating that most trees were detected even when predicted boxes did not fully match the reference delineations. The mean IoU of matched detections was 0.765, indicating generally good spatial agreement despite reduced performance under stricter matching criteria.

**Table 2:** Detection performance of the Faster R-CNN teacher model at varying IoU thresholds.

IoU@	TP	FP	FN	Precision	Recall	F1	Avg IoU
**0.3**	6373	3559	1706	0.641	0.788	0.707	0.697
**0.4**	5851	4081	2228	0.589	0.724	0.649	0.732
**0.5**	5191	4741	2888	0.522	0.642	0.576	0.765

Abbreviations: IoU, intersection over union; TP, true positives; FP, false positives; FN, false negatives; Avg IoU, mean intersection over union of matched detections.

#### Genus classification

Among the evaluated classifiers, ResNet-101 achieved the best performance and was therefore selected for the teacher ensemble (Table 3). The accuracy levels that we obtained indicate that genus-level information can be recovered from aerial crown patches, although confusion remains among spectrally and morphologically similar deciduous genera. The comparison of the genus models is described in Supplementary Note 2.1.

**Table 3:** Genus label quality prior to human curation.

Model	Top-1Acc	Top-5Acc	Precision	Recall	F1	Macro F1
ResNet101	66.11%	94.54%	67.24	66.11	0.658	0.607

Abbreviations: Top-1Acc, top-1 accuracy; Top-5Acc, top-5 accuracy; Macro F1, macro-averaged F1-score.

### Pseudo-label curation outcome and annotation quality

The teacher workflow produced 373,178 pseudo-labels across 143 parent tiles, of which 168,254 annotation instances in 2,965 overlapping subtiles were selected for curation. After removal of duplicates caused by subtile overlap, the remaining teacher predictions were matched against the Greehill terrestrial survey inventory to incorporate survey-derived crown geometries and genus labels before HITL review. Following curation, the released annotation layer contained 117,678 curated tree annotations; assignment to overlapping subtiles yielded 158,086 training instances. Because the two annotators worked on spatially non-overlapping areas, inter-annotator agreement could not be quantified. Although aggregate curation changes could be reconstructed from the attributes of the curated annotation layer, the extent of manual review was not quantified; therefore, correction rates among reviewed annotations could not be estimated (Supplementary Note 1).

### Validation of the released tree inventory

#### Crown-localisation reliability

We compared the auxiliary one-class tree detector with the final ten-class genus detector used for the released inventory (Table 4). At IoU = 0.5, the one-class detector achieved mAP = 0.647, compared with 0.382 for the ten-class detector. This difference shows that locating crowns is more reliable than jointly locating and assigning genera, and that a substantial share of error in the final inventory arises from taxonomic ambiguity rather than failure to identify trees.

**Table 4:** Comparison of tree detection and genus detection performance of the YOLO11l student model.

Task	mAP@0.3	mAP@0.4	mAP@0.5	mAP @0.5–0.95	Precision	Recall	F1	Genus Macro F1
**Tree**	0.754	0.712	0.647	0.345	0.684	0.632	0.657	—
**10-Genus**	0.427	0.409	0.382	0.210	0.465	0.385	0.421	0.627

Abbreviations: ImgSz, input image size; Ch, number of input channels; mAP, mean Average Precision; mAP@0.5–0.95, COCO-style mean Average Precision averaged over IoU thresholds from 0.5 to 0.95; F1, F1-score.

#### Genus classification

**Fig. 6:**
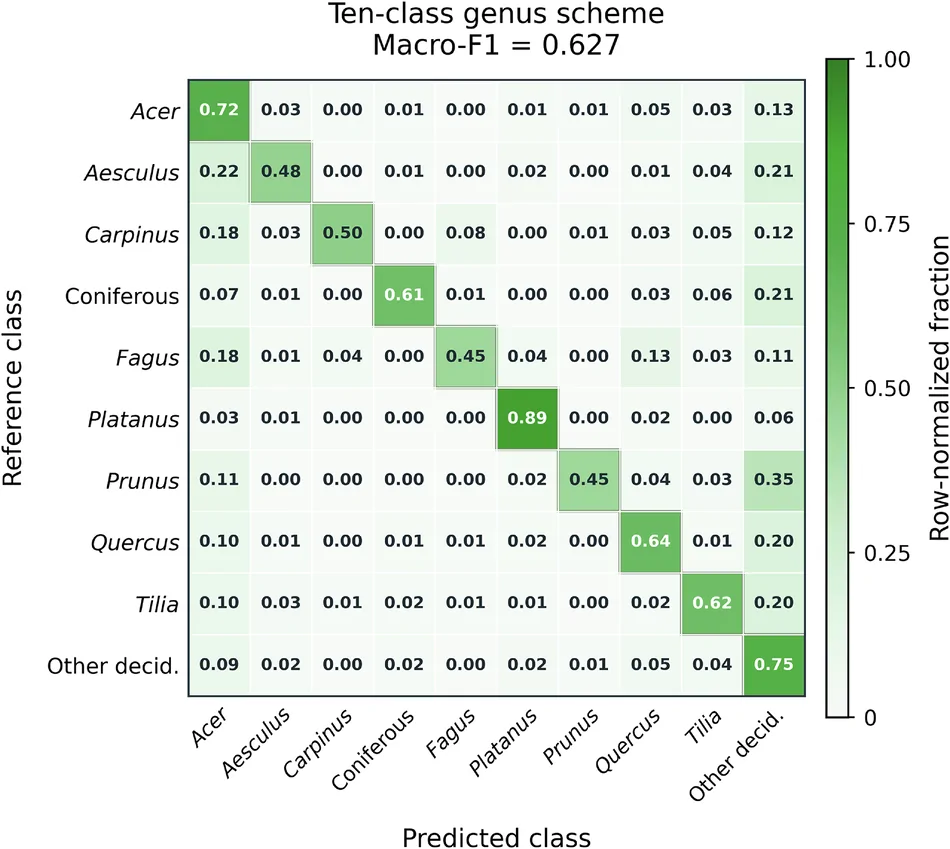
Confusion matrix of ten-class genus-label assignment.

The ten-class model achieved a genus macro F1 of 0.627, while class-specific F1 scores ranged from 0.454 to 0.872 (Table 5). The confusion matrix (Fig. 6) shows that most errors involved confusion among deciduous genera and the “Other Deciduous” class, consistent with similarity in crown appearance. Accordingly, genus labels are better suited to aggregated compositional analyses than to high-confidence interpretation of individual trees.

**Table 5:** Class-specific performance of the 10-class taxonomic scheme with a confidence threshold of 0.3.

Class	Reference instances	Predicted instances	TP	FP	FN	Precision	Recall	F1
*Acer*	2,295	2,348	1,660	688	635	0.707	0.723	0.715
*Aesculus*	622	499	300	199	322	0.601	0.482	0.535
*Carpinus*	74	60	37	23	37	0.617	0.500	0.552
*Fagus*	71	70	32	38	39	0.457	0.451	0.454
*Platanus*	910	950	811	139	99	0.854	0.891	0.872
*Prunus*	126	107	57	50	69	0.533	0.452	0.489
*Quercus*	743	811	473	338	270	0.583	0.637	0.609
*Tilia*	1,259	1,046	776	270	483	0.742	0.616	0.673
Coniferous	556	473	340	133	216	0.719	0.612	0.661
Other Deciduous	3,007	3,299	2,244	1,055	763	0.680	0.746	0.712
**Genus Macro F1**	—	—	—	—	—	**0.649**	**0.611**	**0.627**
**Total**	**9,663**	**9,663**	**6,730**	**2,933**	**2,933**	—	—	—

#### Geographic reliability

The geographically independent city-to-city split described in the Methods evaluated transfer to Freiburg, which contributed no training data.

**Fig. 7:**
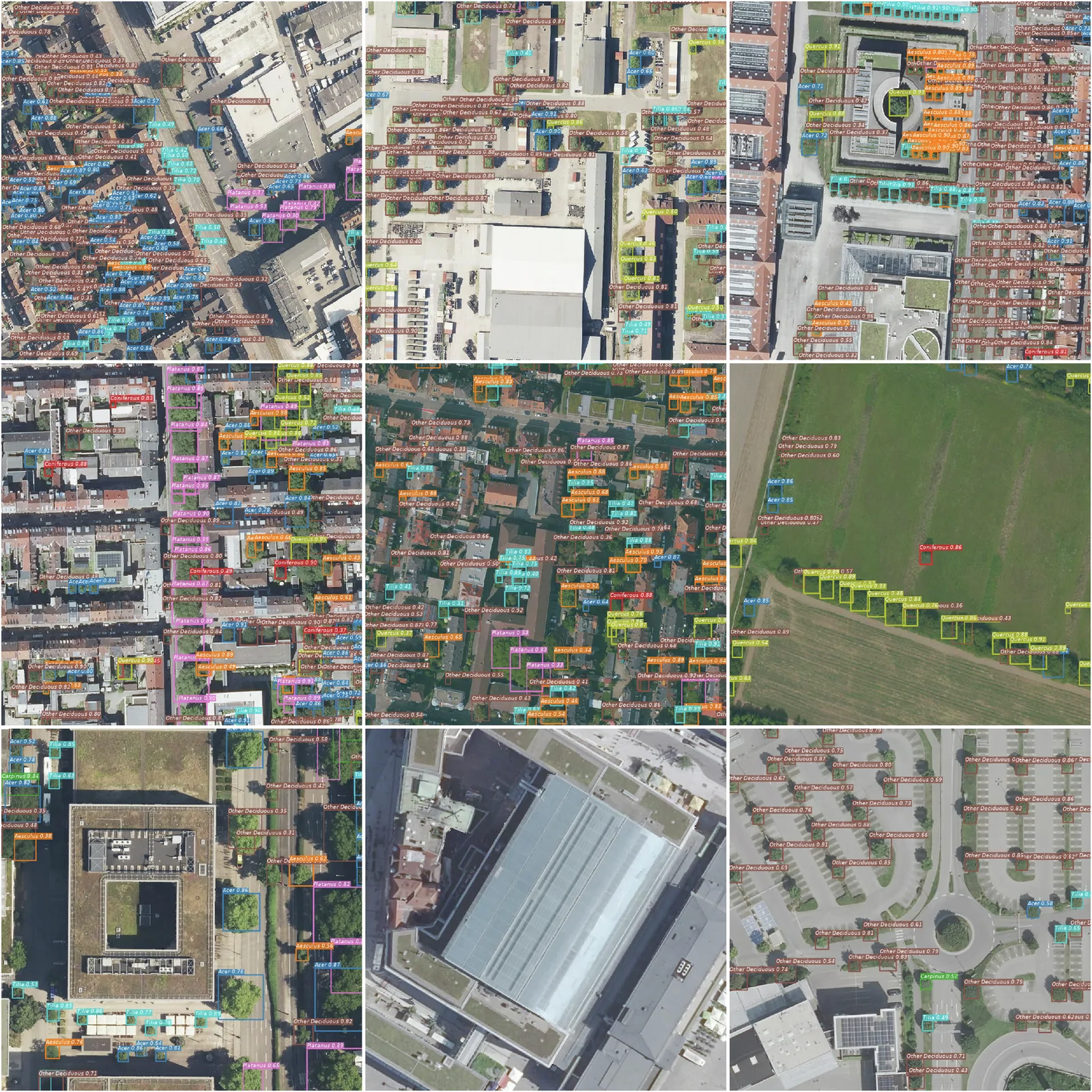
Qualitative examples of predicted genus and confidence across settlement and land-use contexts, including urban cores, residential areas, parks, peri-urban/rural areas, parking lots, and rooftop vegetation.

Qualitative examples of predictions across different settlement types, including dense urban cores, residential neighborhoods, and parks, are shown in Fig. 7. Performance decreased under the city-to-city setting compared with the pooled tile-based split across Mannheim, Karlsruhe, and Freiburg (Table 6), with mAP@0.5 declining from 0.382 to 0.167 and genus macro F1 from 0.627 to 0.215. Precision decreased from 0.465 to 0.425, whereas recall decreased from 0.385 to 0.180, indicating a shift toward more conservative predictions. Overall, genus-level performance was lower under the city-to-city setting, indicating that the dataset is more reliable for aggregated compositional analyses than for high-confidence genus assignment of every individual tree.

**Table 6:** Accuracy of the released inventory under geographic transfer, evaluated on the test partition of each split.

Split	mAP@0.3	mAP@0.4	mAP@0.5	mAP@0.5–0.95	Precision	Recall	F1	Genus Macro F1
Tile-based split	0.427	0.409	0.382	0.210	0.465	0.385	0.421	0.627
City-to-city split	0.219	0.194	0.167	0.106	0.425	0.180	0.254	0.215

Abbreviations: ImgSz, input image size; Ch, number of input channels; mAP, mean Average Precision; mAP@0.5–0.95, COCO-style mean Average Precision averaged over IoU thresholds from 0.5 to 0.95; F1, F1-score.

This asymmetry between precision and recall is informative about the likely source of the decline: if performance had degraded uniformly due to a generally harder recognition task in Freiburg, both precision and recall would be expected to fall together, whereas a large recall drop alongside comparatively stable precision instead suggests that the model’s decision threshold for a given genus is tied in part to visual context specific to the training cities, such as canopy density, planting arrangement, and surrounding street or building morphology, rather than exclusively to genus-intrinsic crown features.

### Bias and uncertainty

Residual class bias is most evident for *Acer*, which is both well represented in the training data and visually similar to several other deciduous genera. Classification uncertainty is greatest for small or partially occluded crowns, mixed plantings, densely built street environments, and other settings in which individual crowns are difficult to separate. The spectral and spatial information available from the 0.2 m RGB + NIR orthophotos and the associated height layer also limits discrimination among morphologically and spectrally similar deciduous genera, including *Acer*, *Tilia*, *Fagus*, and *Carpinus*. An additional source of uncertainty arises from the older LiDAR-derived height data used for initial weak-label generation. nDSM A, derived from DSM1 and DTM1 data acquired between 2016 and 2021, may predate the 2024 imagery by several years. The temporal mismatch may therefore have affected the initial crown delineation where trees were planted, removed, or substantially changed between acquisitions. All teacher-model inputs, student-model training, and statewide inference instead used nDSM B, whose surface information is aligned with the 2024 imagery. This reduces temporal mismatch in the released inventory, although nDSM B has lower vertical accuracy than nDSM A. Users should therefore interpret genus labels cautiously in these settings. Where possible, predictions should be compared with local tree inventories or evaluated through sensitivity analyses using alternative confidence thresholds or broader genus groupings.

### Final inference coverage

The statewide tiling grid comprised 36,674 parent tiles of 1 km × 1 km. After application of the Baden-Württemberg boundary and non-forest mapping mask, 36,653 tiles remained within the prediction domain. Predictions intersecting excluded polygons were removed before release. Quantitative validation was limited to the selected reference survey areas and does not establish uniform statewide accuracy.

## Usage Notes

The dataset comprises a statewide individual-tree inventory and derived spatial summaries of trees outside forests across Baden-Württemberg. Record 1 provides the main tree-level product, including mapped crown geometry, location, estimated height, prediction of genus, and model confidence. Record 6 provides aggregated indicators derived from Record 1 at municipality level, including predicted tree counts, tree density, genus richness, and mean normalized Shannon diversity.

**Fig. 8:**
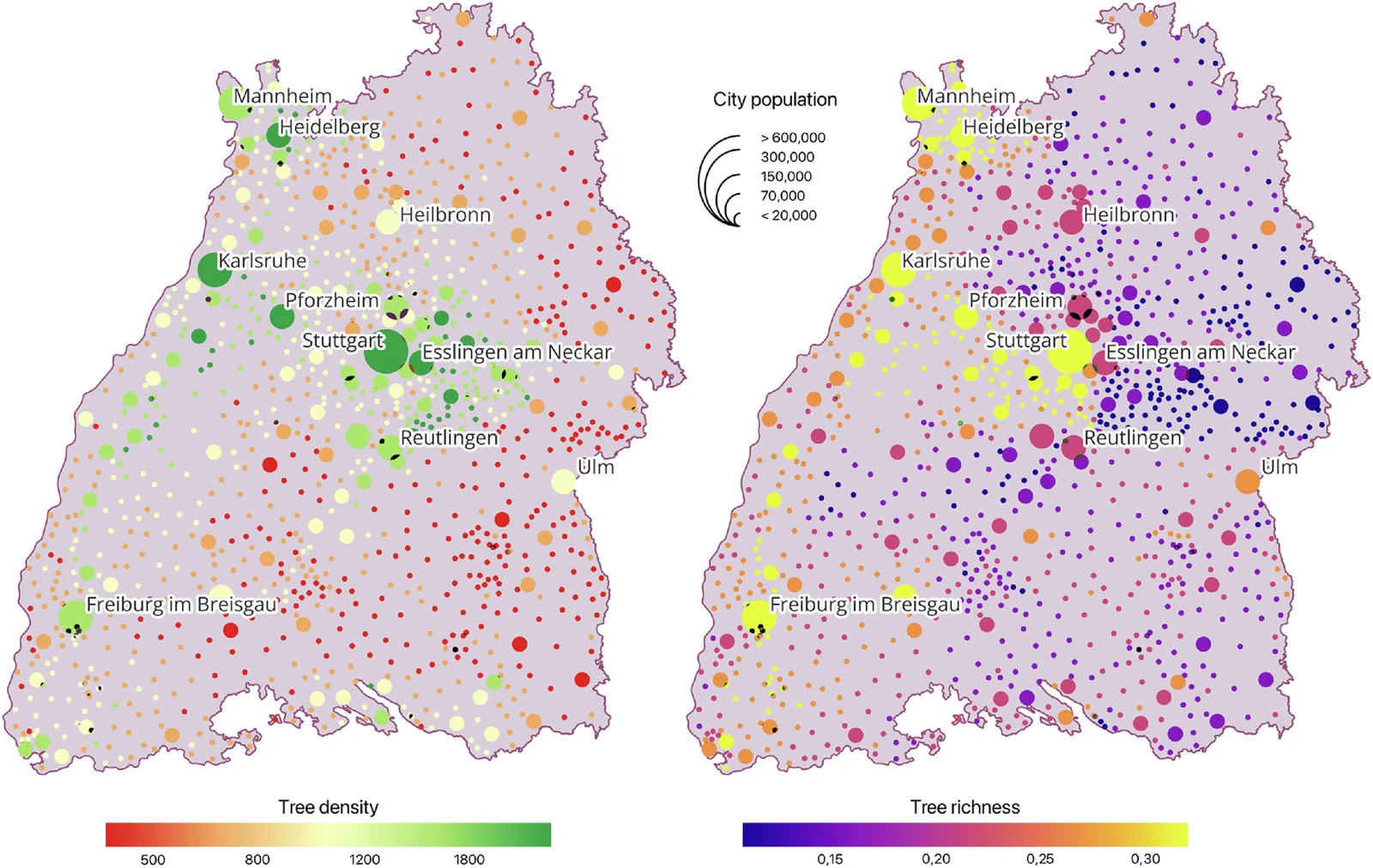
Statewide maps: tree density and tree richness at municipal scale.

Basic descriptive statistics are summarized in Table 7 and Fig. 8, including total tree counts, state-level class frequencies, and tree density (trees per km^2^) for the ten most populous municipalities. Additional examples of potential applications are provided in Supplementary Note 4.

**Table 7:** Tree inventory indicators for the ten most populated municipalities in Baden-Württemberg.

Rank	Municipality	Population	Trees detected	Area analyzed (km^2^)	Tree density (trees/km^2^)	Tree richness
1	Stuttgart	607,543	329,896	156.95	2,101.9	0.361
2	Mannheim	312,654	223,047	125.70	1,774.4	0.397
3	Karlsruhe	304,894	240,754	125.89	1,912.4	0.374
4	Freiburg im Breisgau	232,317	117,184	86.63	1,352.7	0.358
5	Heidelberg	153,659	124,580	63.65	1,957.2	0.420
6	Pforzheim	132,981	98,666	46.51	2,121.3	0.396
7	Heilbronn	128,064	77,172	84.86	909.4	0.213
8	Ulm	124,330	88,154	93.64	941.5	0.262
9	Reutlingen	116,397	94,616	66.91	1,414.0	0.198
10	Esslingen am Neckar	93,726	77,329	34.01	2,273.9	0.207
Total	Baden-Württemberg	10,923,090	16,330,519	21,661.89	753.88	0.208

Tree density was calculated relative to the valid mapped non-forest area. Mean normalised Shannon diversity was derived from intermediate 100 × 100 m grid-cell values as described in the Methods; these intermediate grid-cell data are not included in Record 6.

### Good practice and caveats

Users should keep several limitations in mind when interpreting the data. First, the distinction between forest and non-forest areas in this dataset follows the Basis-DLM land-cover classification and should not be interpreted as an independently delineated ecological boundary. Users should therefore exercise particular caution near forest edges and in dense or transitional vegetation, including wooded parks, orchards, riparian strips, hedgerows, and peri-urban mosaics, where assignment to forest or non-forest may depend on mapping conventions and polygon boundaries. In addition, predictions were classified as settlement or non-settlement TOF using the Basis-DLM *AX_Ortslage* polygons. Because the reference surveys covered selected streets, residential areas, parks, and road corridors in Mannheim, Karlsruhe, and Freiburg, predictions in non-settlement landscapes represent an extrapolation beyond the principal reference-data domain. These predictions are retained and flagged in the dataset but should be interpreted more cautiously, particularly in dense or overlapping vegetation and with respect to genus classification.

Second, the elevation and imagery sources were not perfectly synchronous during initial weak-label generation: nDSM A, derived from DTM1 and DSM1 data acquired between 2016 and 2021, was used for height-based crown delineation, whereas all teacher-model inputs, student-model training, and statewide inference used nDSM B, whose surface information is aligned with the 2024 imagery. nDSM B has an approximate vertical accuracy of 40–60 cm, compared with the higher nominal vertical accuracy of the LiDAR-derived source. Because the initial crown delineation used a 2 m height threshold, temporal and vertical uncertainty may have affected the inclusion of small or young trees close to this threshold. Tree-height values and detections of low-stature vegetation should therefore be interpreted with particular caution.

Third, the statewide inventory represents tree crowns visible in overhead aerial imagery rather than a complete stem inventory. Small trees may be detected when their crowns are exposed and spatially distinct, whereas understory trees and trees beneath larger crowns are likely to be omitted. In dense orchards, allotment gardens, tree-lined avenues, and other settings with overlapping canopies, individual crowns may also be missed, merged, or incorrectly split. Tree counts in such environments should therefore be interpreted as estimates of aerially visible individual crowns rather than complete stem counts.

Fourth, the ten-class taxonomic scheme comprises eight genus classes and the aggregate classes Coniferous and Other Deciduous. Classification uncertainty remains among visually similar broadleaved genera, including *Acer*, *Tilia*, *Fagus*, and *Carpinus*, and between individual genera and the Other Deciduous class. Genus-level labels also do not resolve variation among species within the same genus and should therefore not be interpreted as indicating uniform phenological or other ecological characteristics. The released inventory additionally incorporates a heuristic post-processing rule for *Acer*. Predictions initially assigned to *Acer* with confidence values from 0.30 to below 0.50 were retained as tree detections but reassigned to Other Deciduous, whereas predictions with confidence values of at least 0.50 retained the *Acer* label. The 0.50 threshold was introduced empirically after statewide inference produced an unexpectedly high proportion of *Acer* predictions and was not formally optimised against an independent validation dataset. In addition, the model was trained using summer imagery only and therefore does not explicitly account for seasonal variation in crown appearance. Taxonomic labels are consequently better suited to aggregated compositional analyses than to high-confidence identification of individual trees, and abundance estimates for *Acer* and Other Deciduous should be interpreted with particular caution. For analyses requiring greater taxonomic certainty, users should consider sensitivity analyses based on confidence thresholds, aggregation of visually similar deciduous classes, and comparison with independent local inventories where available.

Finally, model performance is substantially stronger for tree detection than for the ten-genus model (Supplementary Note 2.4). Accordingly, analyses based on the locations, crown geometry, and counts of aerially visible trees are better supported than analyses requiring precise genus assignment. Genus labels are best used for aggregated compositional analyses rather than as high-confidence labels for individual trees. This distinction is particularly important for cross-city generalisation, where genus-level performance declined markedly in the city-to-city evaluation, and genus labels should therefore be interpreted more cautiously outside conditions similar to the training data. Adapting the workflow to other regions should not be assumed to be fully automated, because adaptation to different imagery, environmental conditions, or genus compositions may require targeted local curation and independent validation. Possible strategies for such adaptation are discussed in Supplementary Note 3.

## Supplementary information

Supplementary Information

## Data Availability

The final dataset and the curated HITL-verified annotation dataset have been deposited in heiDATA under a CC BY 4.0 licence. The dataset is publicly available at https://doi.org/10.11588/DATA/MKZPUY^40^.

## Code availability

The custom code used for data preprocessing, model training, evaluation, and statewide inference is archived with the dataset in the heiDATA repository at https://doi.org/10.11588/DATA/MKZPUY. The archived code includes the configuration files, environment specifications, and scripts corresponding to the workflow described in this study and is provided under the AGPL-3.0 licence. The same codebase is maintained at https://github.com/GIScience/tree-genera-mapping; the archive deposited in heiDATA is the fixed version corresponding to this Data Descriptor.
