# Supplementary material for: A genus-labeled dataset of individual trees outside forests in Baden-Württemberg, Germany
Source: Sci Data. 2026 Sep 30;13:1364. doi: 10.1038/s41597-026-08400-y (PMC13627677; doi:10.1038/s41597-026-08400-y)
Supplement: Supplementary file 1 [file 41597_2026_8400_MOESM1_ESM.docx]

**Supplementary Information for *A genus-labeled dataset of individual trees outside forests in Baden-Württemberg, Germany***

**Authors**

Yulia Grinblat^1,6*^, Heike Tost^2,3^, Anastasja Benedyc^2,3^, Andreas Meyer Lindenberg^2,3,^, Somidh Saha^4,5^, Alexander Zipf, ^1,6,7^, Nir Fulman^1,6*^

### **Affiliations**

1. Heidelberg Institute for Geoinformation Technology: HeiGIT, Heidelberg, Germany

2. Department of Psychiatry and Psychotherapy, Central Institute of Mental Health, Medical Faculty Mannheim/Heidelberg University, Mannheim, Germany

3. German Center for Mental Health (DZPG), Partner Site Mannheim Heidelberg Ulm, Mannheim, Germany

4. Research Group Sylvanus, Institute for Technology Assessment and Systems Analysis, Karlsruhe Institute of Technology, Karlsruhe, Germany

5. Institute of Geography and Geoecology, Karlsruhe Institute of Technology, Karlsruhe, Germany

6. Institute of Geography, GIScience Chair, Heidelberg University, Heidelberg, Germany

7. Heidelberg Center for the Environment (HCE), Heidelberg University, Heidelberg

corresponding author: Nir Fulman ([nir.fulman@uni-heidelberg.de](mailto:nir.fulman@uni-heidelberg.de))

* Equal contribution

# **Supplementary Note 1****: Human-in-the-loop curation protocol**

Further details of the human-in-the-loop procedure described in the Methods are provided here. Given the large number of candidate annotations, manual review was targeted rather than exhaustive. Two annotators reviewed spatially non-overlapping subsets of the annotation data in QGIS Desktop [1], such that no spatial area was assigned to both annotators. Individual annotations were therefore not independently reviewed by both annotators; consequently, no formal disagreement-resolution procedure was applied and inter-annotator agreement could not be quantified. Within each assigned subset, the annotator jointly inspected the high-resolution aerial imagery, corresponding height data, and teacher-generated crown boxes and genus labels. The review focused on three main checks: whether the detected object was a tree, whether the bounding box adequately represented an individual crown, and whether the assigned genus was supported by the available visual evidence.

Genus assessment was based primarily on crown shape, texture, color, phenological appearance, planting context, and the annotator’s local knowledge of the surveyed area. Where the genus could not be assessed sufficiently from the aerial imagery, available Google street-level imagery [2] was visually inspected as an additional source of evidence. These views were used to examine features such as leaf morphology, branching structure, bark, stem form, and planting arrangement. Street-level imagery was used only where a visible tree could be matched reliably to its location in the aerial image. Potential differences in acquisition date, season, visibility, and tree condition were considered during interpretation. The street-level imagery is not redistributed with the dataset.

The teacher ensemble combined Faster R-CNN crown detection with ResNet-101 genus classification and produced 373,178 box-and-class pseudo-labels across 143 parent tiles. A total of 2,965 overlapping subtiles containing 168,254 annotation instances were selected for curation. Because adjacent subtiles overlapped, individual trees could occur in more than one subtile; duplicate annotations arising from this overlap were removed before further processing. The remaining predictions were matched against the Greehill terrestrial survey inventory, which contained 42,194 retained records. This matching identified 42,158 survey-derived crowns within the selected annotation set. For matched trees, the teacher-derived bounding box and predicted genus label were replaced by the survey-derived crown geometry and genus label, while the teacher detection-confidence score was retained. Surveyed trees without a corresponding teacher detection were added directly. The resulting set passed to the annotators comprised 119,143 objects: 42,158 survey-derived crowns and 76,985 teacher predictions without a survey counterpart.

During HITL curation, annotators removed false positives, corrected genus labels, adjusted or redrew crown boxes, and added trees for which no suitable candidate annotation existed. Frequently observed false positives included non-tree structures in railway areas, hedge-like vegetation, vineyard structures, and low vegetation on rooftops. The review rejected 392 survey-derived crowns and 1,982 teacher predictions, corrected 982 teacher-derived genus labels, corrected 1,098 existing crown geometries, and newly delineated 360 crowns (Table S1). Although no contemporaneous per-annotation edit log was retained, these aggregate outcomes were reconstructed from the provenance, geometry, and class attributes of the curated annotation layer. Because the two annotators reviewed different spatial subsets, no annotation was independently assessed by both annotators and inter-annotator agreement could not be quantified.

Following curation, the released annotation layer contained 117,678 annotations, comprising 41,764 survey-derived annotations, 71,513 teacher-derived annotations, and 4,401 annotator-supplied annotations. For model development, the curated annotations were subsequently assigned to overlapping image subtiles. Consequently, individual trees could occur in more than one subtile, resulting in 158,086 tiled annotation instances.

**Table S1.** Composition and curation of the annotation layer

| **Input** | **Passed to review** | **Genus label corrected** | **Crown geometry corrected** | **Rejected** | **Total number, n** |
| --- | --- | --- | --- | --- | --- |
| Greehill terrestrial survey | 42,158 |  | 11 (<0.1%) | 392 (0.9%) | 41,764 |
| Teacher ensemble predictions | 76,985 | 982 (1.3%) | 1,087 (1.4%) | 1,982 (2.6%) | 71,513 |
| Annotator-supplied instances |  |  |  |  | 4,401 |
| **Released annotation layer** | **119,143** | **982 (0.8%)** | **1,098 (0.9%)** | **2,374 (2.0%)** | **117,678** |

## **Supplementary Note 2: Model development and configuration selection**

This note documents the comparisons used to select the final model configuration. All comparisons were evaluated on the pooled tile-based split described in the Methods. They characterise model development and are reported here rather than in Technical Validation, because their primary purpose was configuration selection.

### **2.1. Classifier architecture (teacher stage)**

Two classifier architectures were compared for the teacher ensemble (Table S2). ResNet-101 outperformed ResNet-50 (top-1 accuracy 66.11% against 61.66%; macro F1 0.607 against 0.555) and was adopted.

**Table S2.** Teacher-stage classifier architecture comparison

| **Model** | **Top-1Acc** | **Top-5Acc** | **Precision** | **Recall** | **F1** | **Macro F1** |
| --- | --- | --- | --- | --- | --- | --- |
| ResNet50 | 0.617 | 0.907 | 61.99 | 61.66 | 0.615 | 0.555 |
| ResNet101 | 0.661 | 0.945 | 67.24 | 66.11 | 0.658 | 0.607 |
| Abbreviations: Top-1Acc, top-1 accuracy; Top-5Acc, top-5 accuracy; Macro F1, macro-averaged F1-score. | | | | | | |

**2.2. Input modality**

We compared three input configurations: RGB (3 channels), RGB + NIR (4 channels), and RGB + NIR + Height (5 channels). All experiments were conducted using the pooled tile-based split with an input resolution of 640 × 640 pixels. NIR information alone did not improve performance relative to the RGB baseline and resulted in a small decrease across the reported metrics (Table S3). In contrast, the full five-channel configuration yielded the strongest results overall, increasing mAP@0.5 from 0.553 to 0.628 and F1 from 0.570 to 0.635. Based on this analysis, the five-channel configuration was selected for all subsequent experiments and statewide inference.

**Table S3.** Input-modality comparison for tree YOLO11l detector

| **Input / Variant** | **ImgSz** | **Ch** | **mAP@0.3** | **mAP@0.4** | **mAP@0.5** | **mAP@0.5-0.95** | **Precision** | **Recall** | **F1** |
| --- | --- | --- | --- | --- | --- | --- | --- | --- | --- |
| RGB (baseline) | 640 | 3 | 0.694 | 0.640 | 0.553 | 0.261 | 0.632 | 0.530 | 0.570 |
| RGB + NIR | 640 | 4 | 0.661 | 0.613 | 0.538 | 0.248 | 0.596 | 0.522 | 0.557 |
| RGB+NIR+Height | 640 | 5 | 0.747 | 0.704 | 0.628 | 0.302 | 0.647 | 0.623 | 0.635 |
| Abbreviations: ImgSz, input image size; Ch, number of input channels; mAP, mean Average Precision; mAP@0.5–0.95, COCO-style mean Average Precision averaged over IoU thresholds from 0.5 to 0.95; F1, F1-score. | | | | | | | | | |

**2.3. Input resolution**

To assess the effect of spatial context and object scale, we compared input resolutions of 640 × 640 and 1024 × 1024 pixels using the five-channel configuration. Increasing the input resolution led to consistent improvements in detection performance (Table S4), with mAP@0.5 increasing from 0.628 to 0.647 and mAP@0.5–0.95 from 0.302 to 0.345. Gains were also observed at lower IoU thresholds. Precision and recall increased from 0.647 to 0.684 and from 0.623 to 0.632, respectively, resulting in a higher overall F1-score. Based on this analysis, the 1024 × 1024 configuration was selected for the final model.

**Table S4.** Input-resolution comparison for the 5-channel tree YOLO11l detector

| **ImgSz** | **Ch** | [**mAP@0.3**](mailto:mAP@0.3) | [**mAP@0.4**](mailto:mAP@0.4) | [**mAP@0.5**](mailto:mAP@0.5) | [**mAP@0.5-0.95**](mailto:mAP@0.5-0.95) | **Precision** | **Recall** | **F1** |
| --- | --- | --- | --- | --- | --- | --- | --- | --- |
| 640 | 5 | 0.747 | 0.704 | 0.628 | 0.302 | 0.647 | 0.623 | 0.635 |
| 1024 | 5 | 0.754 | 0.712 | 0.647 | 0.345 | 0.684 | 0.632 | 0.657 |
| Abbreviations: ImgSz, input image size; Ch, number of input channels; mAP, mean Average Precision; mAP@0.5–0.95, COCO-style mean Average Precision averaged over IoU thresholds from 0.5 to 0.95; F1, F1-score. | | | | | | | | |

**2.4.** **Taxonomic-resolution comparison**

We compared three separately trained task formulations using the five-channel input configuration and an input resolution of 1024 × 1024 pixels: one-class tree detection, five-class genus-labelled detection, and ten-class genus-labelled detection. The five-class scheme comprised Acer, Coniferous, Platanus, Tilia, and Other Deciduous. The ten-class scheme corresponded to the final taxonomic classification used for the released statewide inventory.

The one-class tree detector achieved the highest detection performance, with mAP@0.5 of 0.647 and F1-score of 0.657 (Table S5). The five-class model achieved mAP@0.5 of 0.501 and macro F1 of 0.745, whereas the ten-class model achieved mAP@0.5 of 0.382 and macro F1 of 0.627. The decrease in performance with increasing taxonomic resolution reflects the greater difficulty of distinguishing morphologically and spectrally similar tree classes from aerial imagery.

**Table S5.** Comparison of one-, five- and ten-genus class task formulations

| **Task** | **mAP@0.3** | **mAP@0.4** | **mAP@0.5** | **mAP**  **@0.5–0.95** | **Precision** | **Recall** | **F1** | **Genus**  **Macro F1** |
| --- | --- | --- | --- | --- | --- | --- | --- | --- |
| **Tree** | 0.754 | 0.712 | 0.647 | 0.345 | 0.684 | 0.632 | 0.657 | — |
| **5-Genus** | 0.559 | 0.538 | 0.501 | 0.269 | 0.545 | 0.492 | 0.518 | 0.745 |
| **10-Genus** | 0.427 | 0.409 | 0.382 | 0.210 | 0.465 | 0.385 | 0.421 | 0.627 |
| Abbreviations: ImgSz, input image size; Ch, number of input channels; mAP, mean Average Precision; mAP@0.5–0.95, COCO-style mean Average Precision averaged over IoU thresholds from 0.5 to 0.95; F1, F1-score. | | | | | | | | |

The five-class results were obtained from a separately trained model and should not be interpreted as a post hoc aggregation of predictions from the ten-class model. Although the five-class formulation provided higher class-assignment performance, the ten-class configuration was retained for statewide inference because it preserved the greater taxonomic detail required for the released inventory.

Training and validation trajectories for the three separately trained task formulations are shown in Figure S1. Figure S2 shows the corresponding row-normalized confusion matrices for the five- and ten-class formulations.

**Figure S1. Training and validation trajectories for the one-class tree-detection and five- and ten-class genus-detection models at an input resolution of 1024 × 1024 pixels.**

# **Figure S2.** Row-normalized confusion matrices for the (a) 10-class and (b) 5-class schemes

# **2.5.** **Class weighting**

The curated training partition had an imbalanced distribution of genus classes. The most frequent class, “Other Deciduous”, contained 43,962 instances, whereas the least frequent class, *Carpinus*, contained 1,884 instances, corresponding to a maximum-to-minimum frequency ratio of approximately 23.3. *Acer* contained 29,611 instances and was the second most frequent class. The observed overprediction of *Acer* was therefore not a conventional minority-class problem.

Class imbalance occurred at the object-instance level, whereas oversampling would operate at the image-subtile level. Individual subtiles generally contained multiple tree instances and often several genera. Repeating a subtile because it contained an underrepresented genus would therefore also repeat the common genera occurring in that subtile and duplicate the same local geographic context. Image-level oversampling could therefore not selectively balance the object-class distribution and was not applied. In addition, the training subtiles were selected from areas overlapping the Greehill reference surveys, so their class frequencies reflect the local tree composition and survey coverage of those areas rather than the statewide genus distribution.

Instead, we evaluated class weighting, which modifies the contribution of individual object classes to the classification loss. We trained an otherwise identical ten-class YOLO11l model using square-root inverse-frequency class weights. Class frequencies were calculated using only the training partition. For each class $c$, containing $n_{c}$ training instances, the unnormalized weight was calculated as

$$w_{c}^{*}=\frac{1}{\sqrt{n_{c}}}.$$

The weights were then normalized to have a mean of 1:

$$w_{c}=\frac{w_{c}^{*}}{\frac{1}{C}\sum_{j=1}^{C} w_{j}^{*}},$$

where $C=10$ is the number of classes. Square-root inverse-frequency weighting was used as a moderated alternative to direct inverse-frequency weighting, which would assign substantially larger weights to the least frequent classes. The normalized weights were applied as class-specific positive weights in the binary cross-entropy classification loss. All other training settings were kept unchanged.

The weighted model performed worse than the unweighted baseline (Supplementary Table S6). Validation mAP@0.5 decreased from 0.382 to 0.262, precision decreased from 0.465 to 0.290, and recall decreased from 0.385 to 0.314. Square-root inverse-frequency weighting therefore did not improve performance under the tested training configuration, and the unweighted model was retained for statewide inference.

**Table S6.** Comparison of the unweighted and class-weighted ten-class models.

| **Model configuration** | **mAP@0.5** | **Precision** | **Recall** |
| --- | --- | --- | --- |
| Unweighted baseline | 0.382 | 0.465 | 0.385 |
| Weighted model (square-root inverse frequency) | 0.262 | 0.290 | 0.314 |
| Abbreviations: mAP@0.5, mean Average Precision averaged over IoU thresholds at 0.5. | | | |

# **Supplementary Note 3:** **Extensibility and reuse**

The released annotations, model configurations, and archived code provide a basis for adapting the workflow to other geographic regions, imagery sources, or taxonomic schemes. Transfer is expected to be most direct where the target data have spatial resolution, spectral bands, height information, acquisition conditions, and genus composition comparable to those used in this study. Direct application under substantially different conditions should therefore be accompanied by local validation and, where necessary, model adaptation.

In the present study, the teacher-generated predictions selected for model development underwent substantial targeted human review before being used as curated reference annotations. The teacher models reduced the effort required to delineate and label trees from scratch by providing initial crown boxes and genus assignments, but they did not eliminate the need for expert curation. The demonstrated scalability of the workflow therefore concerns primarily the automated generation of the statewide inventory after model training, rather than fully automated creation of training data in a new region.

For transfer to another region, manual effort could potentially be reduced through a more selective semi-supervised adaptation strategy. An existing teacher model could first be applied to a large volume of unlabelled target imagery. High-confidence predictions could be retained as pseudo-labels, whereas manual review could be concentrated on a smaller, geographically representative subset and on predictions with low confidence, uncommon classes, conflicting model outputs, or image conditions not represented in the original training data. The reviewed annotations and automatically retained pseudo-labels could then be combined to fine-tune a student model. Pseudo-labelled observations could also be assigned lower training weights than manually reviewed annotations to account for their greater uncertainty.

Such a strategy could reduce annotation effort relative to exhaustive review of the teacher predictions, but it would introduce a greater risk of propagating systematic teacher errors into the student model. An independently reviewed target-region validation and test set would therefore remain necessary to assess transfer performance, calibrate confidence thresholds, and identify class-specific or geographically structured errors. This selective pseudo-labelling strategy was not evaluated in the present study and is presented as a possible extension of the released workflow.

Further extensions could include seasonal or multi-year imagery to capture phenological differences, additional spectral information, and time-series vegetation indices. The workflow could also be adapted to combine RGB and near-infrared imagery, height information, and multitemporal features. Extending the current ten-class scheme would require additional labelled reference data for the newly introduced genera or a revised grouping of taxonomically similar classes.

# **Supplementary Note 4: Example applications**

The TOF-URBORETUM dataset supports applications in urban ecology, planning, biodiversity assessment, and environmental health that require individual-tree data rather than aggregate greenness measures [3]. For example, it can be used to:

- Quantify urban forest structure and composition at tree level, including genus diversity, crown size proxies, and height distributions, for cities and municipalities.
- Conduct environmental justice and urban equity analyses that consider not only canopy cover but also tree size, diversity, and the presence of vulnerable low-diversity assemblages.
- Support resilience and risk assessments by mapping monocultures or genus clusters that may be susceptible to pests, pathogens, or heat and drought stress.
- Enhance environmental health studies by operationalizing taxa-specific exposures (e.g. allergenic genera, pollen sources) instead of relying solely on NDVI or total canopy cover [4, 5].

# **References**

1. QGIS Development Team. *QGIS Geographic Information System*. Open Source Geospatial Foundation Project. <https://qgis.org/> (accessed 12 December 2025).
2. Google. *Google Street View*. <https://www.google.com/streetview/> (accessed 5 December 2025).
3. Forstliche Versuchs- und Forschungsanstalt Baden-Württemberg. *City Tree Suit*. <https://www.citytreesuit.de/> (accessed 5 December 2025).
4. Zhao, T. *et al.* Urban greenspace under a changing climate: Benefit or harm for allergies and respiratory health? *Environmental Epidemiology* **9**, e372, <https://doi.org/10.1097/EE9.0000000000000372> (2025).
5. Tost, H. *et al.* Neural correlates of individual differences in affective benefit of real-life urban green space exposure. *Nature Neuroscience* **22**, 1389–1393, <https://doi.org/10.1038/s41593-019-0451-y> (2019).
